# Investigating the impact of different road scenarios on the induction intensity of motion sickness in electric vehicle passengers

**DOI:** 10.3389/fpsyg.2025.1615498

**Published:** 2025-08-01

**Authors:** Bangbei Tang, Bingjie Luo, Yongfeng Ding, Qiuyang Tang, Yingzhang Wu

**Affiliations:** ^1^School of Intelligent Manufacturing Engineering, Chongqing University of Arts and Sciences, Chongqing, China; ^2^Department of Physiology, Army Medical University, Chongqing, China; ^3^China Automotive Engineering Research Institute Co, Ltd., Chongqing, China; ^4^School of Vehicle and Mobility, Tsinghua University, Beijing, China

**Keywords:** motion sickness severity, physiological signals, real-vehicle dynamics measurements, road characteristics, predictive model

## Abstract

**Introduction:**

With the increasing prevalence of electric vehicles, motion sickness has emerged as a critical factor impairing passenger comfort. Current studies relying on simulated driving face limitations in replicating real-road conditions.

**Methods:**

We conducted real-vehicle experiments across six roadway scenarios: one-way left turn (R1), linear acceleration/deceleration (R2), sudden arrest-activation (R3), uphill S-curve (R4), downhill S-curve (R5), and one-way right turn (R6). A synchronized system (BioRadio + vehicle gyroscopes) captured subjective ratings from participants (*n* = 10) and objective data.

**Results:**

Significant changes occurred in mean values of $\mathrm{GS}R_{\text{mean}}$ , $HR_{\text{mean}}$ , RMSSD, and $\mathrm{RES}P_{\text{mean}}$ during motion sickness (*p* < 0.05), while standard deviations ( $\mathrm{GS}R_{\mathrm{SD}}$ , $\mathrm{RES}P_{\mathrm{SD}}$ ) showed no significance. Motion sickness severity ranked as: R4 (8.4) > R5 (7.7) > R3 (6.3) > R2 (4.4) > R1 (2.0) > R6 (1.4), confirming S-curves as the primary trigger.

**Discussion:**

The logistic regression model achieved 81.25% accuracy in predicting motion sickness states. This study provides empirical evidence for optimizing vehicle motion control and road design to enhance passenger comfort.

## 1 Introduction

Electric vehicles are in a critical period of industrial upgrading and change (Martínez-Díaz et al., 2019), and it has been suggested that SAE Level 5 vehicles may enter the consumer market within the next few decades (Kyriakidis et al., 2015; Wadud et al., 2016). With the development of autonomous driving technology, the role of the driver is gradually shifting to that of a passenger, and research on issues such as ride comfort is becoming increasingly emphasized (Bellem et al., 2018; Zhang et al., 2021). Research shows that in autonomous vehicles, 40.25% of passengers engage in non-driving activities such as reading, texting, or entertainment (Diels, 2014). Among them, 14% often experience motion sickness, and 17% show moderate to severe symptoms of motion sickness. The incidence of motion sickness is 17.24% higher compared to traditional vehicles (Clément et al., 2023; Diels, 2014; Keshavarz and Golding, 2022). Schmidt et al. (2020) conducted an online survey of 4,479 participants from Brazil, China, Germany, the United Kingdom, and the United States. The survey found that 46% of participants had experienced motion sickness in the past 5 years, with China having the highest incidence rate at 61.7%, ranking first among all countries surveyed.

Motion sickness, accompanied by symptoms such as nausea, headache, rapid heartbeat, and vomiting, significantly impairs the riding experience of passengers (Reason, 1978; Cullen, 2019; Zhang et al., 2016). Its occurrence mechanism mainly involves sensory conflict theory and postural instability theory. The sensory conflict theory posits that when visual input conflicts with vestibular and proprioceptive information, the brain struggles to effectively integrate these conflicting signals, leading to discomfort and adverse reactions (Chua et al., 2023). The postural instability theory (Riccio and Stoffregen, 1991; Weech et al., 2018; Smart et al., 2023; Bailey et al., 2022) proposes that motion sickness arises from the effort or failure of the body to maintain postural stability when responding to motion perturbations, with a decline in postural control capabilities constituting the central factor. Building upon the postural instability theory, Smart et al. (2002) established a direct theoretical link between measurements of postural dynamics and the onset of motion sickness through empirical research, and validated the utility of these measurements for quantifying postural instability.

Currently, researchers both domestically and internationally have conducted various studies on motion sickness (Zheng et al., 2023). Research conducted by Li et al. (2019) indicates that when acceleration exceeds 1.50 m/s^2^ or deceleration falls below −0.75 m/s^2^, passengers are significantly more likely to experience motion sickness discomfort. Ren and Zhou (2023) conducted a study using a six-degree-of-freedom riding simulator and found that driving mode and passenger sensitivity to motion sickness significantly affect the severity of motion sickness. They also established a correlation model between brain oxygenation signals and the degree of motion sickness. Keshavarz et al. (2022) developed a model using galvanic skin response, electrocardiogram, respiration, and subjective responses, which can explain 41% of the variance in the subjective questionnaire scores for motion sickness symptoms (FMS). Wang et al. (2020) established a comfort prediction model based on vehicle motion and passenger physiological parameters, achieving an accuracy rate of 84%. Barabino et al. (2019) collected and analyzed passengers’ evaluations of motion sickness discomfort to develop a comfort detection system specifically for buses. This system focuses on assessing passengers’ motion sickness experiences while riding, effectively identifying the factors that impact passenger comfort. Additionally, it provides important insights for optimizing the quality of public transportation services, ultimately enhancing passengers’ overall riding experience and satisfaction. Yusof et al. (2020) developed a wearable anti-motion sickness system that uses a smart vibrating sleeve worn on the passenger’s wrist. This device precisely triggers multi-frequency vibration waveforms during turns and other driving conditions to provide tactile feedback. The experimental results demonstrate its effectiveness in preventing motion sickness. However, research on motion sickness often employs simulated driving environments in the laboratory, failing to adequately account for the various complex dynamic factors present in real-world road conditions.

Common assessment methods for motion sickness include subjective and objective evaluation techniques. Subjective assessments primarily rely on the passengers’ personal feedback regarding their discomfort. Commonly used subjective scales include: The Motion Sickness Questionnaire (MSQ) (Kennedy et al., 1965) requires participants to rate 20 to 30 symptoms based on their level of discomfort, ranging from no symptoms to vomiting. The Motion Sickness Susceptibility Questionnaire (MSSQ) (Golding, 1998) is used to assess a passenger’s susceptibility to motion sickness. The Misery Scale (MISC) (Bos et al., 2005) uses a rating system from 0 to 10 to assess discomfort based on the current state of the individual. In this study, the MSSQ was used to screen passengers for susceptibility to motion sickness, while the MISC assessed the level of motion sickness symptoms in real-time during the experiment.

Human physiological signals, as spontaneous responses of the body, are less likely to be influenced by the subjective awareness of passengers, resulting in good accuracy and scientific validity (Barabino et al., 2019; Beggiato et al., 2019; Tang et al., 2024; Wang et al., 2019). Nobel et al. (2012) identified skin conductance response as a measure of motion sickness and noted an inherent relationship between physiological signals such as sweating and body temperature and the occurrence of motion sickness. Radhakrishnan et al. (2020) explored the effects of different autonomous driving modes on passenger comfort using heart rate variability (HRV) and galvanic skin response (GSR). The results indicated that galvanic skin response (GSR) is the optimal indicator for assessing passenger comfort. Liu et al. (2020) induced motion sickness using a driving simulator and collected electroencephalogram (EEG) signals. The results indicated that EEG data can effectively assess virtual-induced motion sickness (VIMS) and reveal significant individual differences in susceptibility to motion sickness. Galvanic skin response (GSR) (Yu et al., 2020), heart rate (HR) (Deng et al., 2024), and respiratory rate (RESP) (Shin et al., 2022) are easy to collect and cost-effective (Gao et al., 2021; Diels et al., 2016), making them suitable for motion sickness research in real driving scenarios. Therefore, this study uses skin conductance, respiratory rate, and heart rate as indicators to assess changes in motion sickness levels. Evaluating motion sickness solely from a subjective or objective dimension poses certain limitations. Therefore, this study employs a combination of subjective and objective assessment methods.

This study aims to analyze the sensitivity differences in the levels of motion sickness induced by six types of road scenarios: one-way left turn (R1), linear acceleration and deceleration (R2), sudden arrest-activation (R3), uphill S-curve (R4), downhill S-curve (R5), and one-way right turn (R6). This study aims to analyze the physiological mechanisms based on autonomic nervous responses and to develop a quantitative assessment model for the severity of motion sickness using multimodal physiological features. The ultimate goal is to quantify motion sickness levels, mitigate the severity of motion sickness, and enhance passenger comfort. Therefore, this study primarily addresses the following issues:

(1) What changes occur in skin conductance, respiratory rate, and heart rate as a result of motion sickness?
(2) Is there a correlation between objective physiological data and subjective levels of motion sickness? If so, what is the nature of this correlation?
(3) Is there a significant difference in the impact of six different road conditions on the severity of motion sickness?

## 2 Materials and methods

**Figure 1:**
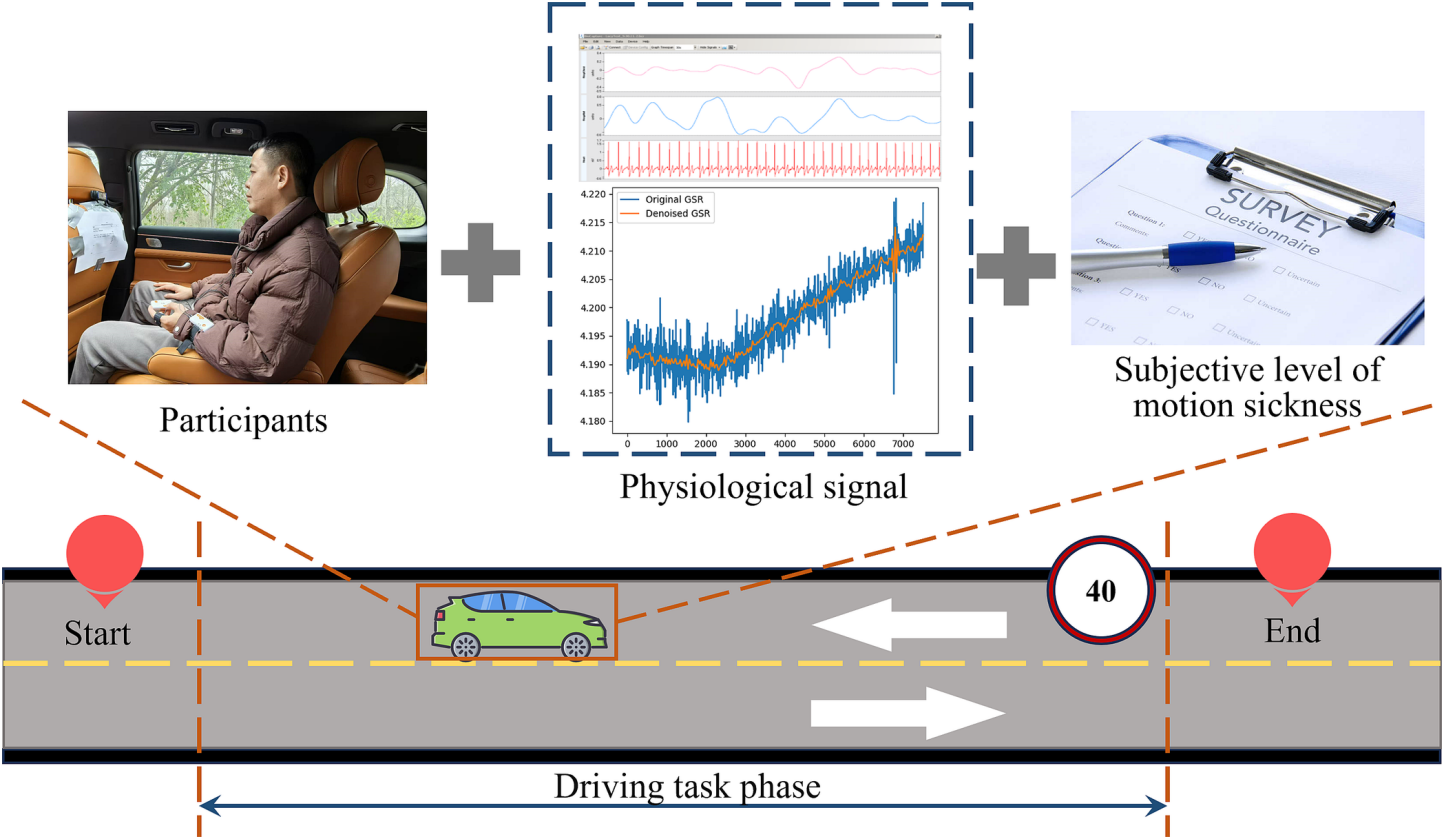
Schematic diagram of the experimental process.

This study employed a real-vehicle testing method, dividing six types of road scenarios into six task points. As participants traveled through these task points, they were asked to rate their level of motion sickness discomfort at each point. Throughout the process, physiological data from the passengers were continuously collected. The data were segmented using a time-marking method, followed by further analysis of both subjective and objective data to quantify the severity of motion sickness under the six different road scenarios (as shown in Figure 1).

### 2.1 Participant

Research indicates that the incidence of motion sickness is higher in females than in males (Schmidt et al., 2020). Therefore, this study recruited 10 participants (6 females and 4 males) through a paid recruitment process, with an average age of 23.7 years (standard deviation = 4.53). All experimental procedures and contents of this study were approved by the Ethics Committee of Chongqing University of Arts and Sciences (Approval No.: CQWL202540), and the handling of participants’ data complied with the Declaration of Helsinki. The screening process was carried out in three rounds: the first round aimed to ensure the physical health of each participant, excluding those with cold symptoms, a history of vestibular dysfunction, or otolith disorder. The second round involved a subjective test using the Motion Sickness Susceptibility Questionnaire (MSSQ), requiring a total score of ≥6 to confirm that participants were susceptible to motion sickness. The third round excluded participants who had abnormal routines, consumed alcohol, or engaged in vigorous physical activity within the past 24 h. After obtaining the participants’ consent, the research team signed informed consent forms with the participants, informing them of the experimental content and the tasks they were required to complete during the study.

### 2.2 Experimental scenario

**Figure 2:**
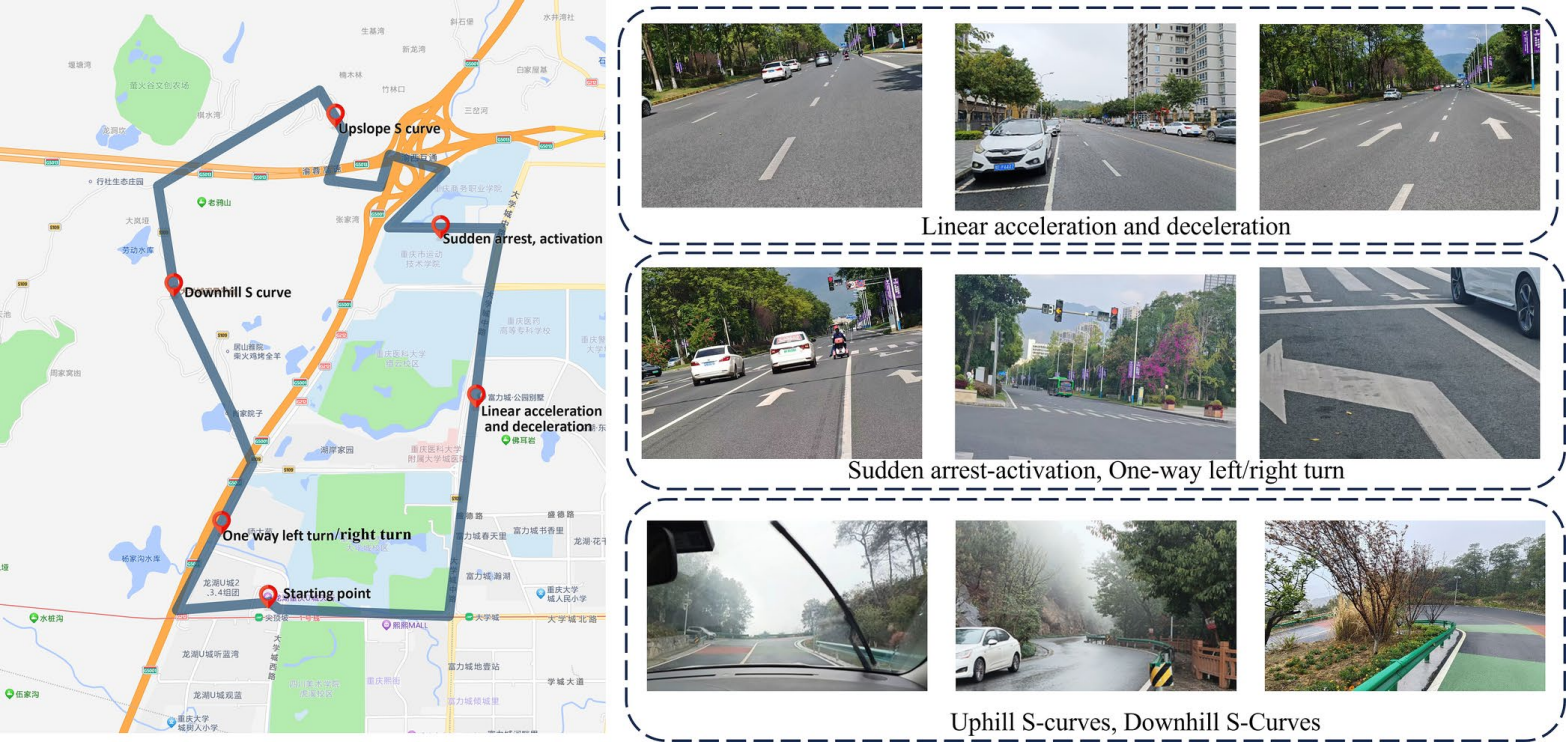
Experimental route and real road scenarios.

To ensure the safety of the experiment and the authenticity of the road scenarios, the trial site was selected on the roads of the university town. The test route includes typical straight sections, one-way turns, and S-shaped curves to meet the needs of the experimental testing. The total distance of the journey is 13 kilometers, with the driving speed at the task points set to 40 km/h, and the estimated driving time is approximately 40 min (as shown in Figure 2). Before the experiment begins, drivers need to familiarize themselves with the site in advance to ensure smooth and steady transitions between different road scenarios.

**Figure 3:**
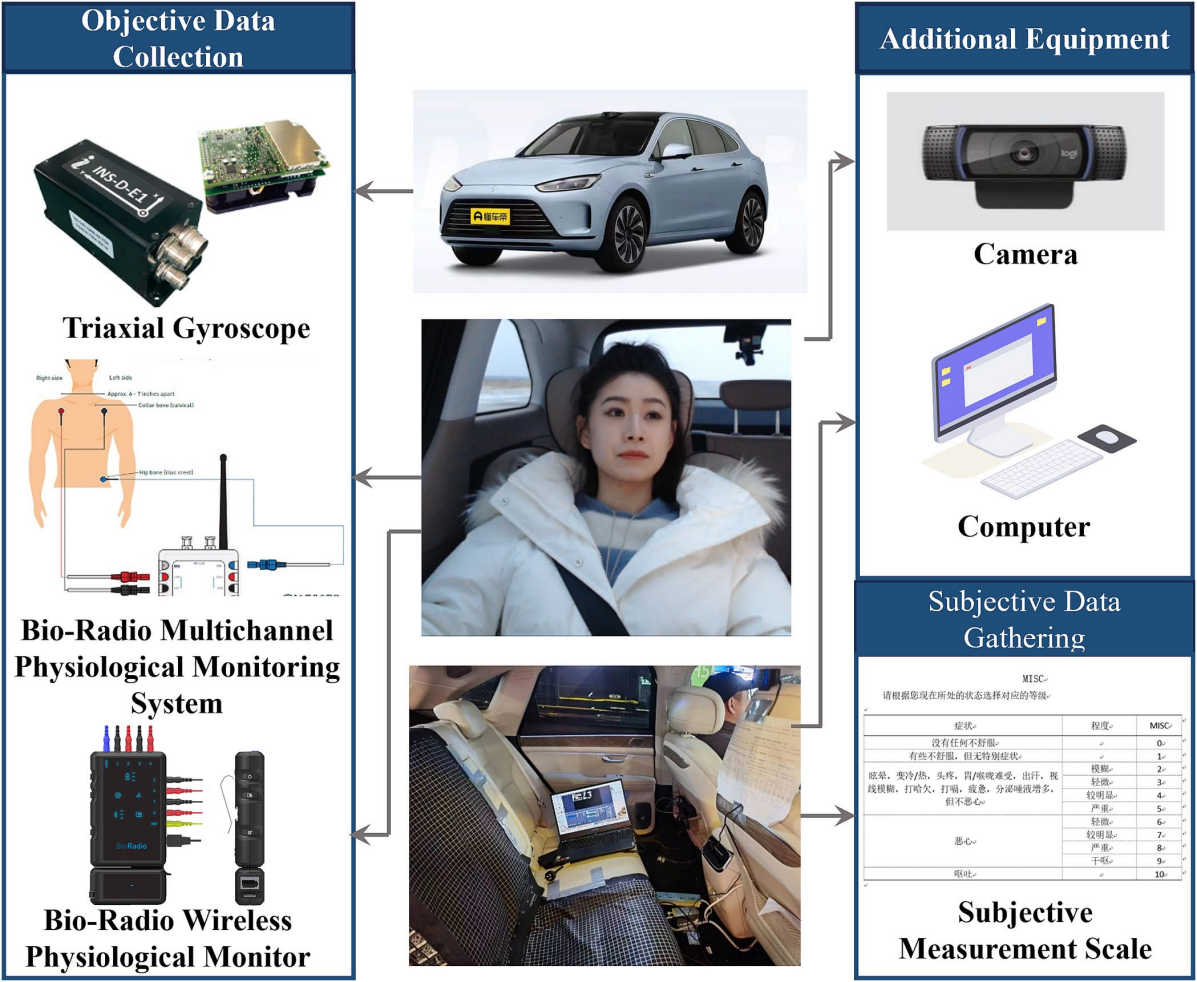
Construction of a multimodal data collection experimental platform for motion sickness in real vehicles.

The experimental scenario setup (as shown in Figure 3) involves a 5-seat automatic electric vehicle. The participant is seated in the right side of the second row. The required equipment includes:

(1) A three-axis gyroscope (data update frequency: 200 Hz; speed accuracy: 0.03 m/s) records the vehicle’s state parameters throughout the entire experiment.
(2) A multi-channel physiological recorder (featuring 8 single-module channels with a sampling frequency of 250 Hz) gathers data on the participant’s skin conductance, electrocardiogram, respiration, and other physiological metrics.
(3) A high-definition camera system captures the content of participant interviews; the MISC scale is used to assess participants’ motion sickness levels. Additional equipment, including data cables and a laptop, is utilized for data collection, transmission, storage, and subsequent analysis.

### 2.3 Experimental procedure

The experiment is divided into three phases: the preparation phase, the experimental implementation phase, and the completion phase. In the preparation phase, the lead investigator first verifies the participants’ basic information, then provides a detailed explanation of the experiment’s specific content and procedures, and assists the participants in wearing the experimental equipment.

**Figure 4:**
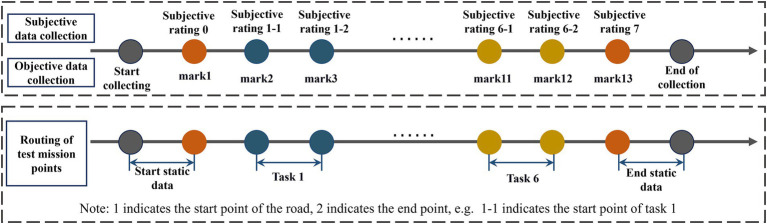
Schematic diagram of experimental data collection process.

During the experimental implementation phase, the vehicle will sequentially travel to six task points. Before and after each task point, the lead investigator will collect participants’ MISC subjective motion sickness ratings through a question-and-answer format and will mark the task points in the multi-channel physiological monitoring system. At the beginning of the experiment, the vehicle will proceed to the starting point of the road, where participants will adjust to a calm state and gather 60 s of physiological data as a baseline. Following this, the vehicle will successively visit the six task points. Throughout the process, objective data will be continuously recorded and categorized based on task points and silent state markers; time stamping will be used to precisely capture data for different road conditions. At the end of Task 6, participants will collect 60 s of final state data (as shown in Figure 4). After the experiment concludes, the lead investigator will assist the participants in removing the experimental equipment and conduct in-depth interviews.

## 3 Data processing and analysis

### 3.1 Subjective data preprocessing and feature extraction

Data preprocessing was conducted on the raw subjective data to obtain valid analytical data: First, different road condition segments were precisely extracted using trial time alignment methodology; this was followed by data cleaning procedures, including the removal of outliers (Based on the Interquartile Range (IQR) method for outlier detection, with thresholds set at Q1–1.5 × IQR and Q3 + 1.5 × IQR, data points exceeding this range are flagged as outliers). Missing values are filled using the mean interpolation method based on adjacent data points. Finally, the data was standardized using Z-score standardization. After the above process, 80 sets of valid subjective data were obtained. The mean, maximum, minimum, and standard deviation of the subjective data for each task phase represent the signal values of that phase. The calculation process of the signal values is as follows Equations (1–4):


(1) $$\bar{M}=\frac{1}{n}\sum_{i=1}^{n}M_{i}$$

(2) $$M_{\max}=\max(M_{1},M_{2},\cdots ,M_{n})$$

(3) $$M_{\min}=\min(M_{1},M_{2},\cdots ,M_{n})$$

(4) $$M_{\sigma }=\sqrt{\frac{1}{n}\sum_{i=1}^{n}{\left(M_{i}-\bar{M}\right)}^{2}}$$

In the formula: *n* is the number of data points, $M_{i}$ is the *i*th data point.

### 3.2 Comparative analysis of subjective assessment data

The collected raw physiological signals are often contaminated by noise and artifacts, necessitating preprocessing. In this study, a multimodal physiological signal processing approach was employed for the specific preprocessing of galvanic skin response (GSR), heart rate (HR), and respiratory (RESP) signals. The GSR signal was decomposed into multiple frequency sub-bands using discrete wavelet transform (DWT), followed by soft thresholding for denoising, and the processed sub-bands were ultimately reconstructed into a complete signal through wavelet inverse transform. The HR signal underwent high-pass filtering with a cutoff frequency of 0.5 Hz to eliminate low-frequency noise and extract time-domain feature data. Additionally, the RESP signal was processed with a band-pass filter at a cutoff frequency of 0.1 Hz to extract the time-domain waveform.

Based on the six task nodes in the experimental process, the physiological signals collected synchronously were segmented, and feature parameters for each frequency band were extracted. For the galvanic skin response signal, the mean skin conductance level ( $\mathrm{GS}R_{\text{mean}}$ ) and the standard deviation of skin conductance level ( $\mathrm{GS}R_{\mathrm{SD}}$ ) were extracted to represent the GSR values for that phase. For the heart rate signal, the average heart rate ( $HR_{\text{mean}}$ ) and the root mean square of successive differences (RMSSD) of the RR intervals were extracted to represent the heart rate values for that phase. For the respiratory signal, the mean respiratory frequency ( $\mathrm{RES}P_{\text{mean}}$ ) and the standard deviation of respiratory frequency ( $\mathrm{RES}P_{\mathrm{SD}}$ ) were extracted to represent the respiratory signal values for that phase. The difference between the calm phase and the task phase is used to calculate the signal change value. The calculation processes for each signal value are as follows Equations (5–10):


(5) $$\mathrm{GS}R_{\text{mean}}=\frac{1}{n}\sum_{i=1}^{n}{\mathrm{GSR}}_{i}$$

In the formula: $\mathrm{GS}R_{\text{mean}}$ represents the mean skin conductance level during a specific phase, ${\mathrm{GSR}}_{i}$ denotes the skin conductance value at the *i*th phase, and *n* represents the number of sampling points.


(6) $$\mathrm{GS}R_{\mathrm{SD}}=\sqrt{\frac{1}{n-1}\sum_{i=1}^{n}{\left({\mathrm{GSR}}_{i}-\mathrm{GS}R_{\text{mean}}\right)}^{2}}$$

In the formula: $\mathrm{GS}R_{\mathrm{SD}}$ represents the standard deviation of skin conductance level during a specific phase.


(7) $$HR_{\text{mean}}=\frac{1}{n}\sum_{i=1}^{n}{\mathrm{HR}}_{i}$$

In the formula: $HR_{\text{mean}}$ represents the average heart rate during a specific phase, ${\mathrm{HR}}_{i}$ denotes the heart rate value at the *i*th phase, and N indicates the number of sampling points.


(8) $$\text{RMSSD}=\sqrt{\frac{1}{n-1}\sum_{i=1}^{n-1}{\left({\mathrm{RR}}_{i}-RR_{i+1}\right)}^{2}}$$

In the formula: RMSSD represents the heart rate variability based on the root mean square of successive differences. ${\mathrm{RR}}_{i}$ indicates the RR interval at the *i*th moment, which is the time interval between heartbeats. $RR_{i-1}$ denotes the RR interval at the (*i − 1*)th moment, representing the duration of the previous heartbeat cycle. RR interval refers to the time interval between two successive R waves on an electrocardiogram.


(9) $$\mathrm{RES}P_{\text{mean}}=\frac{1}{n}\sum_{i=1}^{n}{\text{RESP}}_{i}$$

In the formula: $\mathrm{RSE}P_{\text{mean}}$ represents the mean respiratory rate for a given phase, and ${\text{RESP}}_{i}$ represents the respiratory rate value for the *i*th phase.


(10) $$\mathrm{RES}P_{\mathrm{SD}}=\sqrt{\frac{1}{n-1}\sum_{i=1}^{n}{\left({\text{RESP}}_{i}-\mathrm{RES}P_{\text{mean}}\right)}^{2}}$$

In the formula: $\mathrm{RES}P_{\mathrm{SD}}$ represents the standard deviation of the respiratory rate for a given phase.

### 3.3 Comparative analysis of subjective data

A comprehensive dataset integrating subjective data and objective physiological indicators was constructed. Subsequently, a mapping model between subjective data and objective physiological indicators was developed to enable the quantitative assessment of motion sickness severity across different road environments. The specific formula is Equations (11, 12):


(11) D(t)=F(G(t),H(t),R(t))

Where D(t) is the degree of motion sickness, *F* represents the mapping function, G(t) is the electrodermal activity data, H(t) is the heart rate data, R(t) is the respiratory rate, and *t* is the task phase time.

The selected test route comprised six typical road conditions. Participants subjectively ranked their susceptibility to motion sickness for each condition, with Rank 1 indicating the most motion sickness-inducing scenario and Rank 6 the least. Results are presented in Table 1. Road Condition 4 was identified as the most motion sickness-inducing scenario, ranked first by 50% of participants. Neither Condition 1 nor Condition 6 received any first-place rankings. This indicates that single-direction turns (R1and R6) are less likely to induce passenger motion sickness compared to other typical road conditions.

**Table 1:** The statistical results of susceptibility to motion sickness in road scenarios.

Road scene	Percentage (%)					
	Ranked 1	Ranked 2	Ranked 3	Ranked 4	Ranked 5	Ranked 6
R1	0%	0%	0%	20%	50%	30%
R2	0%	10%	20%	50%	10%	10%
R3	20%	20%	40%	10%	10%	0%
R4	50%	30%	10%	10%	0%	0%
R5	30%	40%	20%	10%	0%	0%
R6	0%	0%	10%	0%	30%	60%

Based on the subjective ranking results of participants’ motion sickness susceptibility across six typical road conditions (Table 1), this study further quantified the induction effect weights of different road types on motion sickness symptoms. The calculation formula is as follows:


(12) $$S=P_{i}\times W_{j}\times 2$$

In the formula: S For motion sickness composite score. $P_{i}$ : The proportion of frequency at which a specific road scenario was rated as “most likely to induce motion sickness” (ranked 1) by occupants. (Exact percentage values are provided in Table 1.) $W_{j}$ : denotes the weighting factor, with rank-based weights assigned as follows: Rank 1 (most motion sickness-inducing) = 5 points, Rank 2 = 4 points, Rank 3 = 3 points, Rank 4 = 2 points, Rank 5 = 1 point, Rank 6 (least motion sickness-inducing) = 0 points. Normalization Factor 2: Convert the comprehensive score to a 0–10 scale, aligning with the MISC scale range to enhance result comparability. The final motion sickness comprehensive score S ranges from 0 to 10, where higher scores indicate greater likelihood of the road condition inducing motion sickness symptoms in occupants.

### 3.4 Comparative analysis of objective biometric datasets

This study employs the Kruskal-Wallis non-parametric test to analyze the statistical differences in physiological signal distributions among different levels of motion sickness. Furthermore, it utilizes partial η^2^ (Partial Eta Squared, η^2^_p_) to quantify the explanatory power of each physiological indicator regarding motion sickness severity, thereby assessing their sensitivity. Finally, Pearson correlation analysis is used to validate the consistency between subjective motion sickness ratings and objective physiological changes.

### 3.5 Prediction of motion sickness severity in different road scenarios

A logistic regression prediction model was established to validate the effectiveness of physiological signal changes in predicting motion sickness severity induced by different road conditions. In the predictive model, physiological data indicators were used as independent variables, while the subjective MISC scale score (0–10 points) was converted into a binary dependent variable based on the motion sickness threshold (MISC > 1), categorizing 0–1 points as “asymptomatic” and 2–10 points as “motion sickness discomfort.” The model’s predictive performance was objectively evaluated by calculating the Area Under the Receiver Operating Characteristic Curve (AUC).

## 4 Results

### 4.1 Comparative analysis results of subjective data

**Figure 5:**
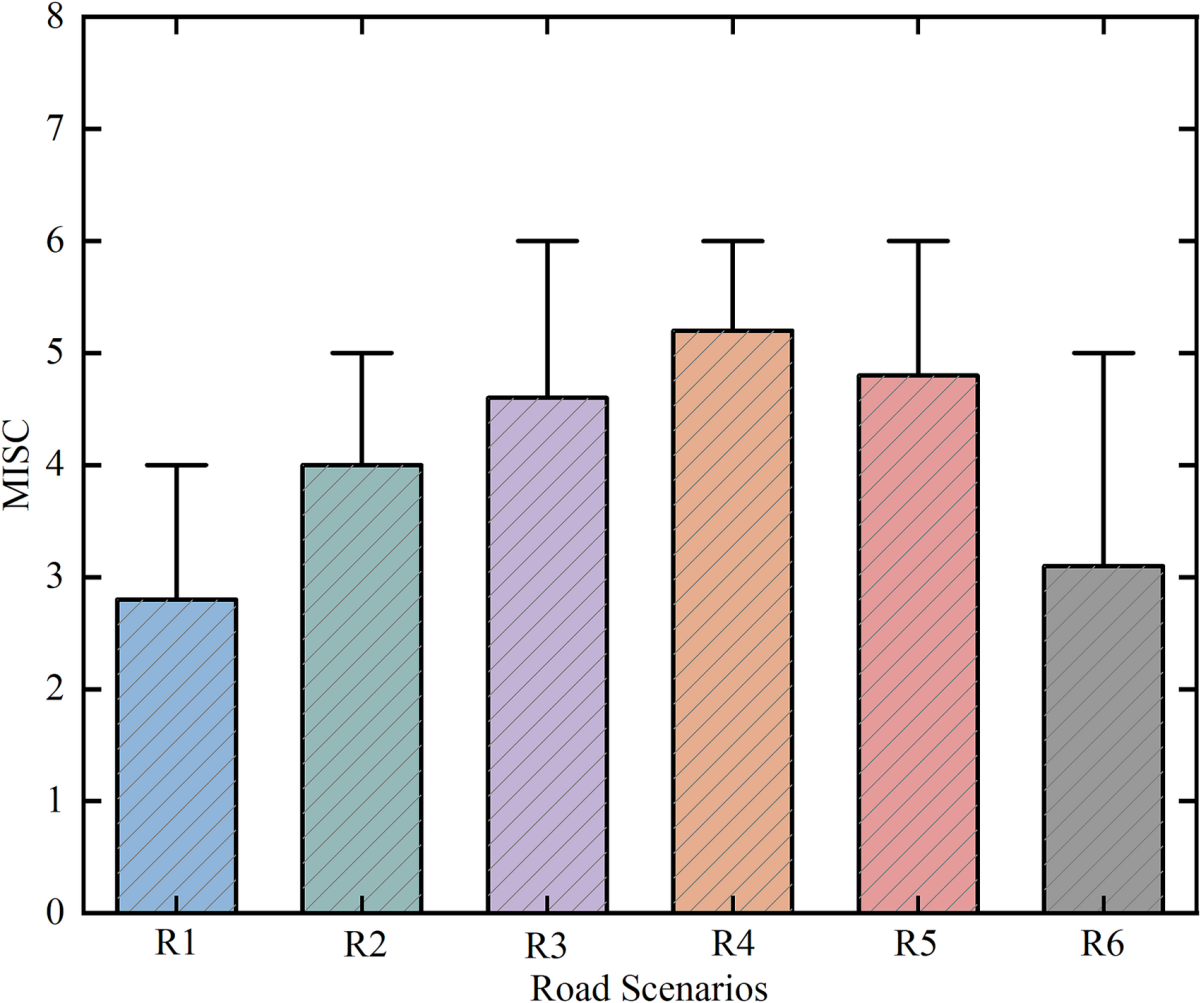
Statistical parameters chart of MISC motion sickness levels for different road types.

Based on the variation data of MISC motion sickness levels across different road scenarios (as shown in Figure 5), a one-way ANOVA was conducted to examine the effect of road type on passengers’ subjective motion sickness levels. Prior to data analysis, the Kolmogorov–Smirnov test was performed to assess normality (*p* > 0.05), followed by a homogeneity of variances test. The results indicated that the mean (W = 0.455, *p* = 0.768), maximum (W = 0.529, *p* = 0.715), minimum (W = 0.508, *p* = 0.730), and standard deviation (W = 0.537, *p* = 0.704) of the subjective motion sickness levels all met the criteria for homogeneity of variances (*p* > 0.05). With confirmation that the data satisfied the assumptions of normality and homogeneity of variances, the one-way ANOVA results demonstrated significant statistical differences in the effects of different road scenarios on passengers’ motion sickness levels (*p* < 0.05).

**Figure 6:**
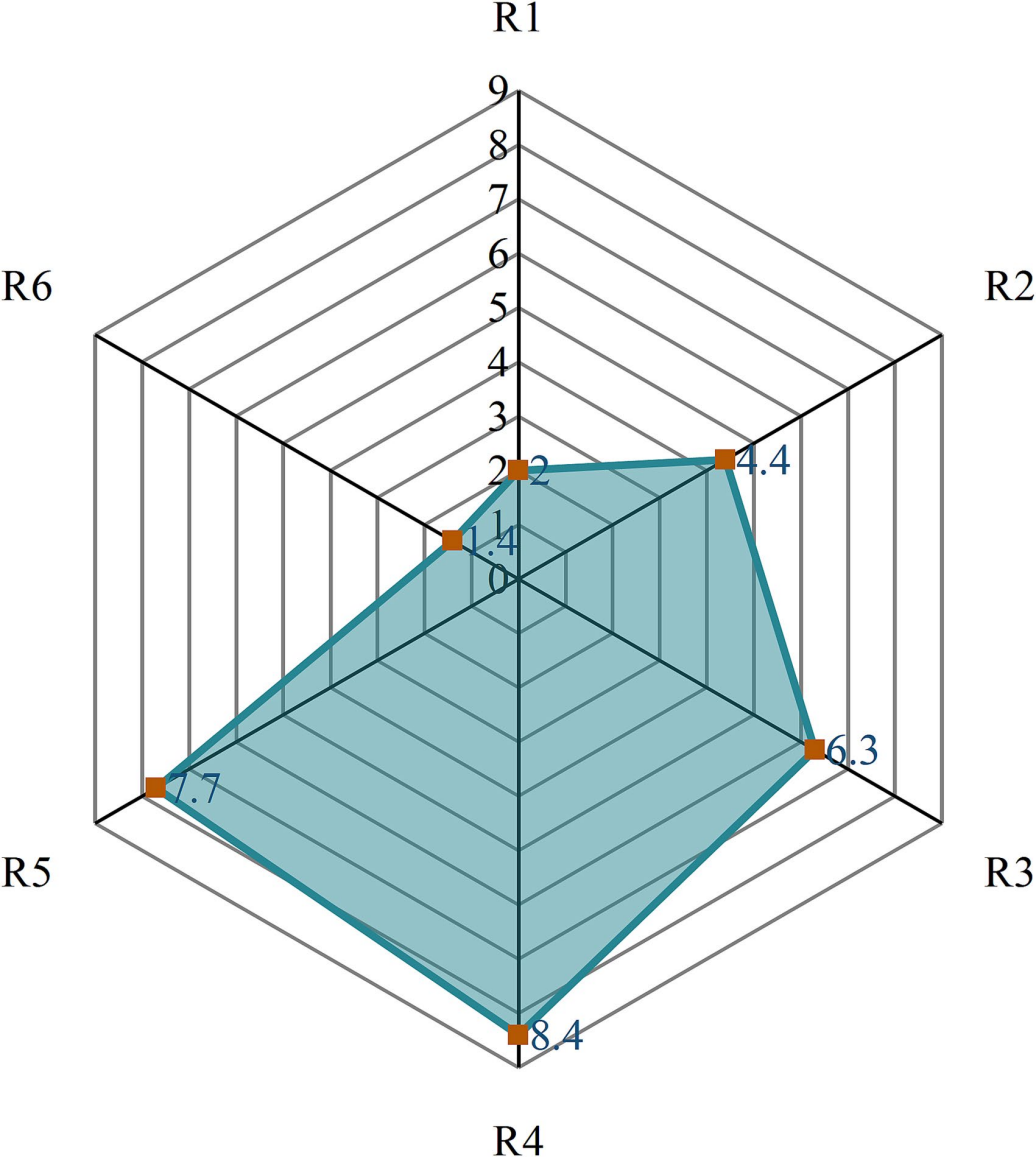
Comprehensive assessment scores for different road types.

The comprehensive calculation results of the induced effects of six types of road conditions on motion sickness are shown in Figure 6. The results indicate that the motion sickness inducing intensity of different roads ranks as follows: R4 > R5 > R3 > R2 > R1 > R6. Among them, R4 has a significantly higher comprehensive motion sickness score (M = 8.4) than the other roads, suggesting that R4 exerts the strongest stimulation on the vestibular system of passengers and is the most likely to induce motion sickness symptoms. In contrast, R6 shows the lowest comprehensive motion sickness score, indicating that R6 has relatively minimal sensitivity to inducing motion sickness.

### 4.2 Comparative analysis results of objective data

In the objective data analysis, we examined the significance and sensitivity of physiological information, including skin conductance, heart rate, and respiratory frequency, among participants, with the results shown in Table 2. Specifically, the indicators $\mathrm{GS}R_{\text{mean}}$ (H = 23.89, *p* < 0.01, η^2^ = 0.070), $\mathrm{GS}R_{\mathrm{SD}}$ (H = 10.19, *p* < 0.05, η^2^ = 0.029), $HR_{\text{mean}}$ (H = 16.25, p < 0.05, η^2^ = 0.048), RMSSD (H = 16.61, p < 0.05, η^2^ = 0.049), and $\mathrm{RES}P_{\text{mean}}$ (H = 21.456, p < 0.05, η^2^ = 0.063) all exhibited significant differences. In contrast, $\mathrm{RES}P_{\mathrm{SD}}$ (H = 3.098, *p* > 0.05, η^2^ = 0.009) did not reach statistical significance, and its very low effect size indicates that this parameter has poor sensitivity to motion sickness responses. Among these indicators, $\mathrm{GS}R_{\text{mean}}$ (η^2^ = 0.070) showed the largest effect size, demonstrating a significant correlation with subjective data and indicating a notable sensitivity to motion sickness. Conversely, although $\mathrm{GS}R_{\mathrm{SD}}$ (η^2^ < 0.04) achieved statistical significance, its small effect size results in relatively limited sensitivity for distinguishing levels of motion sickness. Therefore, this paper will primarily analyze the physiological characteristics of the four parameters: $\mathrm{GS}R_{\text{mean}}$ , $HR_{\text{mean}}$ , RMSSD, and $\mathrm{RES}P_{\text{mean}}$ .

**Table 2:** The results of the statistical analysis of objective physiological data.

Statistic	Physiological measures					
	$\mathrm{GS}R_{\text{mean}}$	$\mathrm{GS}R_{\mathrm{SD}}$	$HR_{\text{mean}}$	RMSSD	$\mathrm{RES}P_{\text{mean}}$	$\mathrm{RES}P_{\mathrm{SD}}$
H	23.89	13.19	16.25	16.61	21.456	3.098
$\eta^{2}$	0.070	0.038	0.048	0.049	0.063	0.009
P	<0.05	<0.05	<0.05	<0.05	<0.05	p > 0.05

**Figure 7:**
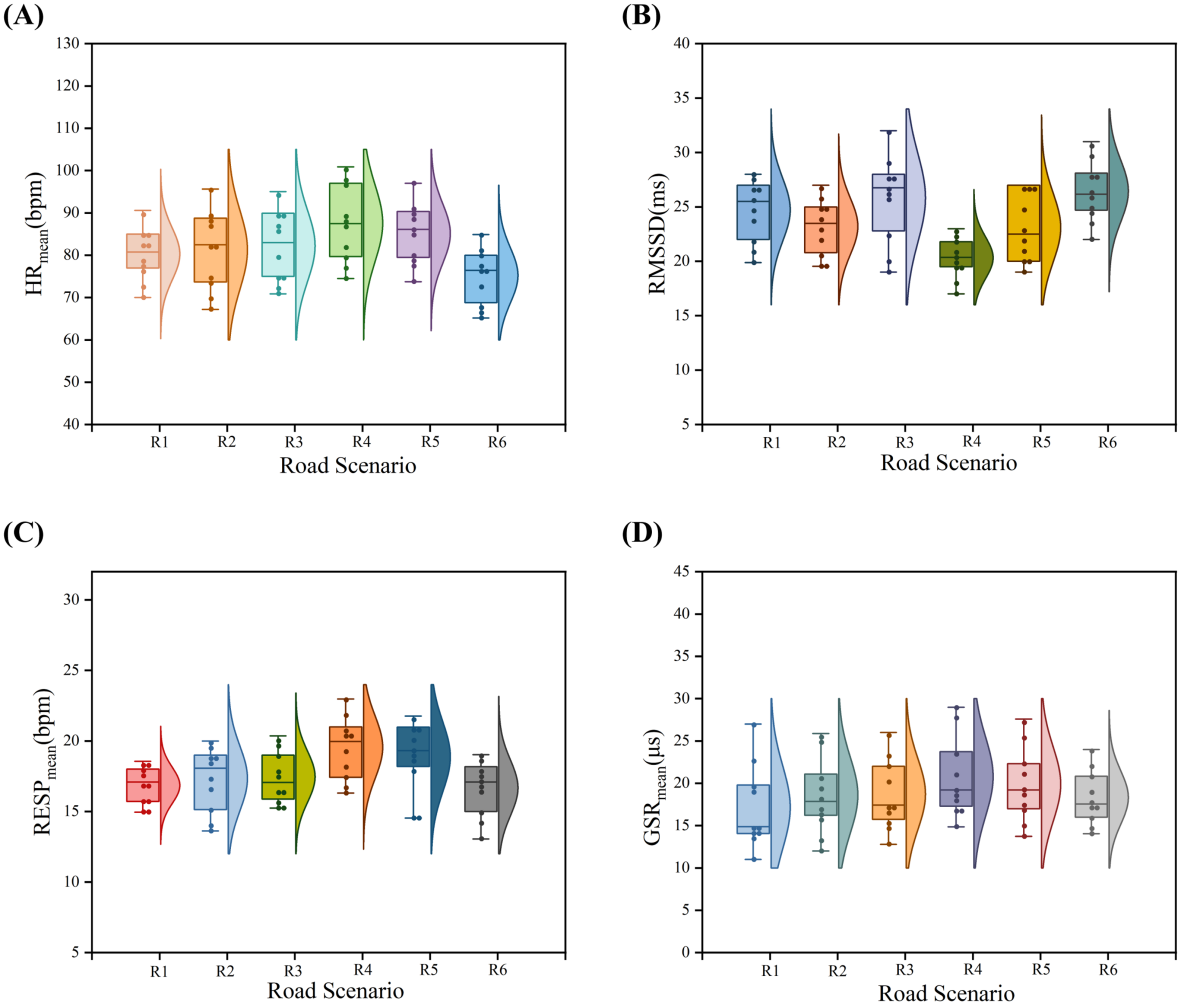
Changes in objective physiological indicators across six different road scenarios. **(A)** Distribution characteristics of skin conductance across six road scenarios; **(B)** Distribution characteristics of heart rate across six road scenarios; **(C)** Distribution characteristics of heart rate variability across six road scenarios; **(D)** Distribution characteristics of respiratory rate across six road scenarios.

The monitoring and data analysis of various physiological indicators reveal significant differences in the induction of motion sickness symptoms across different road conditions. As shown in Figure 7A, the $\mathrm{GS}R_{\text{mean}}$ is significantly higher on road R4 compared to other road conditions, while the values for R1 and R6 are relatively low. The analysis of $HR_{\text{mean}}$ , presented in Figure 7B, indicates that the heart rate values for R4 and R5 are higher, with R4 reaching its peak average heart rate. The results for the RMSSD indicator, depicted in Figure 7C, show a contrasting trend, with higher RMSSD values for R1 and R6, while R4 has the lowest value. Additionally, the $\mathrm{RES}P_{\text{mean}}$ indicator in Figure 7D shows larger values for roads R4 and R5, with R6 exhibiting the lowest value.

The statistical analysis of the objective data indicates that $\mathrm{GS}R_{\text{mean}}$ , $HR_{\text{mean}}$ , and $\mathrm{RES}P_{\text{mean}}$ are positively correlated with subjective motion sickness ratings, while the RMSSD indicator shows a negative correlation. However, all indicators consistently demonstrate that road R4 has the highest sensitivity for inducing motion sickness. In contrast, the motion sickness induced by a left turn (R6) and a right turn (R1) is relatively low, with a smaller range of difference in values.

In Pearson correlation analysis, the absolute value of the correlation coefficient (|*ρ*|) approaching 1 indicates a strong linear association between variables. Table 3 presents the full-sample Pearson correlation analysis results between physiological indicators and subjective motion sickness severity (MISC), demonstrating significant statistical associations (*p* < 0.05) between MISC and galvanic skin response mean ( $\mathrm{GS}R_{\text{mean}}$ ), respiratory rate mean ( $\mathrm{RES}P_{\text{mean}}$ ), heart rate mean ( $HR_{\text{mean}}$ ), and heart rate variability (RMSSD). Specifically, $\mathrm{GS}R_{\text{mean}}$ (*p* = 0.685), $\mathrm{RES}P_{\text{mean}}$ (*p* = 0.629), and $HR_{\text{mean}}$ (*p* = 0.432) exhibited significant positive correlations with MISC, with $\mathrm{GS}R_{\text{mean}}$ demonstrating the strongest association. Conversely, the parasympathetic nervous activity marker RMSSD (*p* = −0.498) showed a significant negative correlation with MISC. The above results clearly confirm that different dimensions of the autonomic nervous system exhibit significantly differentiated response patterns in motion sickness reactions.

**Table 3:** Pearson correlation analysis of occupants’ subjective perception (MISC) with electrodermal activity $(\mathrm{GS}R_{\text{mean}}),$ cardiac parameters $(HR_{\text{mean}},$ RMSSD), and respiratory metrics $\left(\mathrm{RES}P_{\text{mean}}\right)$ in the sample cohort.

Parameters	Pearson correlation coefficient				
	MISC	$\mathrm{GS}R_{\text{mean}}$	$HR_{\text{mean}}$	RMSSD	$\mathrm{RES}P_{\text{mean}}$
MISC	1	0.685^**^	0.432^**^	−0.498^**^	0.629^**^
$\mathrm{GS}R_{\text{mean}}$	0.685^**^	1	0.316^*^	0.003	0.312
$HR_{\text{mean}}$	0.432^**^	0.316^*^	1	−0.342^**^	0.360^*^
RMSSD	−0.498^**^	0.003	−0.342^**^	1	−0.240
$\mathrm{RES}P_{\text{mean}}$	0.629^**^	0.312	0.360^**^	−0.240	1

*Indicates significance at *p* < 0.05.

**Indicates significance at *p* < 0.01.

### 4.3 Prediction results of motion sickness severity based on physiological parameters

**Figure 8:**
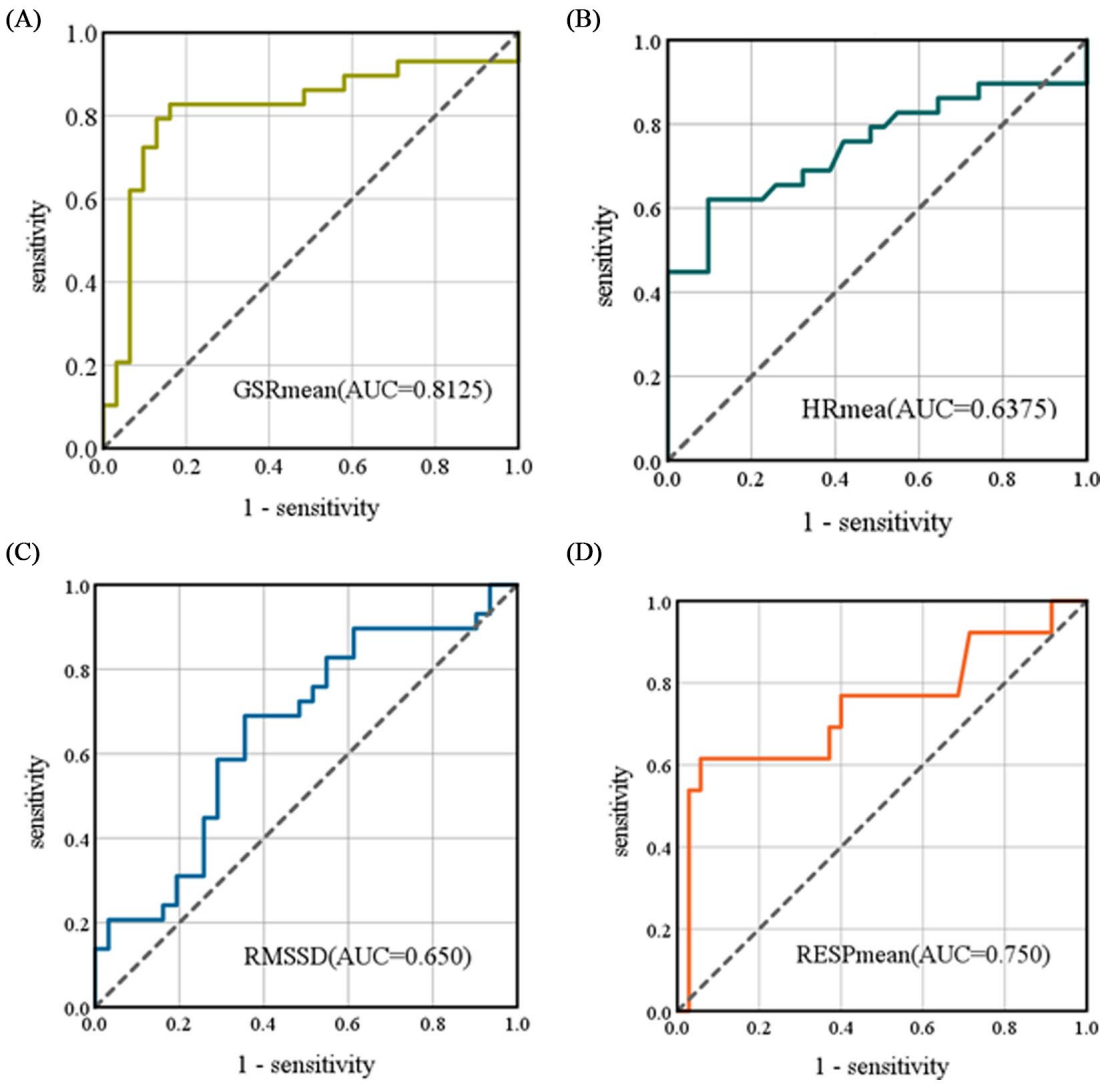
ROC curves for the logistic regression model. **(A)** The ROC curve for $\mathrm{GS}R_{\text{mean}}$ as a univariate input, **(B)** The ROC curve for $HR_{\text{mean}}$ as a univariate input, **(C)** The ROC curve for RMSSD as a univariate input, **(D)** The ROC curve for $\mathrm{RES}P_{\text{mean}}$ as a univariate input. The AUC represents the area under the curve formed by the X and Y axes.

This study developed a logistic regression model to predict motion sickness severity (MISC) using autonomic physiological indicators: galvanic skin response ( $\mathrm{GS}R_{\text{mean}}$ ), heart rate ( $HR_{\text{mean}}$ ), heart rate variability (RMSSD), and respiratory rate ( $\mathrm{RES}P_{\text{mean}}$ ). The model identified significant positive associations between elevated MISC levels and increases in $\mathrm{GS}R_{\text{mean}}$ (*p* < 0.05), $HR_{\text{mean}}$ (*p* < 0.05), and $\mathrm{RES}P_{\text{mean}}$ (*p* < 0.05), while reduced RMSSD (*p* < 0.05) correlated negatively with symptom severity. Univariate predictive performance analysis demonstrated that $\mathrm{GS}R_{\text{mean}}$ achieved the highest discriminative power (AUC = 0.8125) (as shown in Figure 8A), significantly surpassing $HR_{\text{mean}}$ (AUC = 0.6375) (as shown in Figure 8B), RMSSD (AUC = 0.650) (as shown in Figure 8C), and $\mathrm{RES}P_{\text{mean}}$ (AUC = 0.750) (as shown in Figure 8D), with $HR_{\text{mean}}$ and RMSSD showing limited standalone predictive utility (AUC < 0.7). Further variable importance analysis confirmed that GSR had the greatest contribution to the model, with its predictive value clearly superior to that of heart rate and respiratory indicators. Therefore, when $\mathrm{GS}R_{\text{mean}}$ is used as an input for the model, the prediction performance is optimal, achieving an accuracy of 81.25% (as shown in Table 4).

**Table 4:** Confusion matrix of the model under univariate input.

Anticipate			
Data set	Motion sickness	Asymptomatic	Percentage correct
Motion sickness	65	15	81.25%

## 5 Discussion

This study investigates the relationship between the degree of motion sickness and autonomic nervous system responses across different roadway scenarios. The results reveal significant differences in the levels of motion sickness induced by various road conditions, which lead to systematic changes in physiological indicators such as $\mathrm{GS}R_{\text{mean}}$ , $\mathrm{GS}R_{\mathrm{SD}}$ , $HR_{\text{mean}}$ , RMSSD, $\mathrm{RES}P_{\text{mean}}$ , and $\mathrm{RES}P_{\mathrm{SD}}$ . Among these, the trends in $\mathrm{GS}R_{\text{mean}}$ (*p* < 0.05), $HR_{\text{mean}}$ (*p* < 0.05), RMSSD (*p* < 0.05), and $\mathrm{RES}P_{\text{mean}}$ (*p* < 0.05) exhibit statistically significant differences. Although there are inter-group differences in $\mathrm{GS}R_{\mathrm{SD}}$ , its correlation with motion sickness levels is low ( $\eta^{2}$ = 0.029), preventing it from reaching statistical significance. This indicates that changes in $\mathrm{GS}R_{\mathrm{SD}}$ and $\mathrm{RES}P_{\mathrm{SD}}$ cannot effectively distinguish whether an individual is experiencing motion sickness.

When passengers experience mild motion sickness induced by left turns (R1) and right turns (R6), the differences in $\mathrm{GS}R_{\text{mean}}$ , $HR_{\text{mean}}$ , RMSSD, and $\mathrm{RES}P_{\text{mean}}$ across groups are relatively small. However, during moderate or higher levels of motion sickness induced by S-bends, $\mathrm{GS}R_{\text{mean}}$ shows a significant increasing trend. Although $HR_{\text{mean}}$ and $\mathrm{RES}P_{\text{mean}}$ also rise concurrently, their increase is less pronounced. This finding is consistent with the conclusions drawn by Irmak et al. (2021), highlighting that skin conductance responses are highly sensitive to the severity of motion sickness.

Correlation analysis shows that $\mathrm{GS}R_{\text{mean}}$ (r = 0.685, *p* < 0.05), $HR_{\text{mean}}$ (r = 0.432, *p* < 0.05), and $\mathrm{RES}P_{\text{mean}}$ (r = 0.629, *p* < 0.05) exhibit significant positive correlations with subjective motion sickness levels, while RMSSD (r = −0.498, *p* < 0.05) demonstrates a significant negative correlation. The comprehensive analysis of subjective and objective data indicates that the severity of motion sickness induced by six typical roadway conditions is ranked as follows: uphill S-curve (R4) > downhill S-curve (R5) > sudden arrest-activation (R3) > linear acceleration and deceleration (R2) > one way left turn (R1) > one way right turn (R6). Among these, S-bends induce the highest motion sickness effects, likely due to the frequent changes in vehicle heading angle combined with low-frequency lateral oscillations (Xie et al., 2023).

The motion sickness prediction model constructed based on objective physiological indicators demonstrated an accuracy of 81.25%. ROC curve analysis indicated that the predictive efficacy of $\mathrm{GS}R_{\text{mean}}$ (AUC = 0.8125) significantly outperformed other parameters: $\mathrm{RES}P_{\text{mean}}$ (AUC = 0.750), $HR_{\text{mean}}$ (AUC = 0.6375), and RMSSD (AUC = 0.650). Notably, both $HR_{\text{mean}}$ and RMSSD contributed relatively limited predictive power to the model, with AUC values below 0.7, clearly lower than that of $\mathrm{GS}R_{\text{mean}}$ .

However, this study has some limitations. First, the physiological indicators extracted in the study were primarily confined to signals reflecting autonomic nervous activity (GSR, HR, RESP), yet failed to incorporate postural dynamics data that theoretically demonstrates strong predictive relevance. This limitation restricts the examination of a more comprehensive theoretical framework for motion sickness mechanisms. Assessment of motion sickness severity is relatively limited; future research could incorporate other physiological indicators, particularly in combination with high-precision postural kinematics monitoring devices. Second, the age range of participants recruited in this study was not sufficiently broad; future studies should expand the sample size to cover a more diverse population, thereby enhancing the reliability and generalizability of the research. Third, the study employed only logistic regression to build prediction models; future research should explore superior predictive models that integrate multi-source information by synchronously collecting postural dynamics and multimodal physiological data where feasible.

## 6 Conclusion

In summary, this study preliminarily identifies the autonomic nervous system changes (GSR, RESP, and HR) associated with different degrees of motion sickness induced by various roadway conditions, providing valuable insights for future research aimed at alleviating motion sickness. The findings have significant implications for the field of automotive engineering. At the vehicle design level, strategies can be implemented to optimize suspension system stiffness parameters and seat structural dynamics in response to the driving conditions associated with S-bends, effectively attenuating the transmission of vertical vibration energy caused by composite centripetal forces and thereby reducing passenger motion sickness severity. At the level of intelligent driving, the study contributes to the development of path planning algorithms by introducing a motion sickness risk cost function, which can enhance passenger comfort by limiting the rate of curvature change in continuous turns and the frequency of sudden accelerations and decelerations. Future research should expand the sample size (currently *n* = 10) and incorporate synchronized electroencephalogram (EEG) and eye-tracking monitoring to analyze the multi-level regulatory mechanisms of the vestibular, visual, and autonomic nervous systems, thereby establishing a more generalizable model for predicting and intervening in motion sickness.

## Data Availability

The raw data supporting the conclusions of this article will be made available by the authors, without undue reservation.
